# Incidence, duration, and related factors of urinary incontinence in women after childbirth: a systematic review

**DOI:** 10.1590/1806-9282.20241054

**Published:** 2024-12-20

**Authors:** Begum Kirik, Hatice Yildiz

**Affiliations:** 1 Yeditepe University, Faculty of Health Sciences, Department of Nursing – İstanbul, Türkiye.; 2 Marmara University, Faculty of Health Sciences, Department of Obstetrics and Gynecology Nursing, Division of Nursing – İstanbul, Türkiye.

## INTRODUCTION

The International Continence Society (ICS) defines urinary incontinence (UI) as the complaint of involuntary urinary leakage and associated hygiene issues^1^. Postpartum urinary incontinence (PUI) refers to the first occurrence of involuntary urinary leakage during the postpartum period and is prevalent, affecting one in five women who give birth^2^. Some women experience urinary issues during pregnancy, and the recovery process can be prolonged after childbirth^3^. Studies indicate that most cases of PUI, particularly stress urinary incontinence (SUI), resolve within weeks to months or within a year. However, 10–20% of women with PUI may still experience UI issues 5 years after childbirth^4,5,6^. While SUI is the most common type of PUI, urge UI and mixed UI can also occur. Although UI is particularly significant during the postpartum period, it is often overlooked in healthcare^2^. UI, with its multifaceted impact on women after childbirth, has been the focus of numerous prevalence studies. The prevalence of postpartum stress urinary ıncontinence (PSUI) varies due to factors such as sample size, population demographics, follow-up duration, parity, and study methodology^7,8,9,10,11^. Factors during pregnancy and childbirth, such as hormonal changes, increased abdominal pressure from the growing uterus, and pelvic floor stress, are associated with UI^12^. Wang et al. identified risk factors for PUI, including vaginal birth, advanced maternal age, obesity, excessive weight gain, operative birth, episiotomy, and diabetes^13^. PUI significantly challenges women’s quality of life, affecting self-esteem and the mother’s ability to care for her newborn^14^. This systematic review aims to analyze studies on the frequency of UI during the first year postpartum, the most common type of PUI, and the associated factors.

### Research questions

1. What is the incidence of PUI?
2. What are the types of PUI?
3. What factors may cause/are associated with PUI?

## METHODS

### Study design

This systematic review examines the incidence of UI in women during the first year postpartum, focusing on the most prevalent type and associated factors. The study’s methodology and reporting adhere to the PRISMA model^15^.

### Article screening strategy, selection, and exclusion criteria

This retrospective review scanned research articles published in peer-reviewed journals from 2013 to 2023, using search engines such as PUBMED, Science Direct, Scopus, EBSCO, ULAKBİM, and TR-Dizin. Keywords included “postpartum/postnatal/puerperal,” “urinary incontinence,” “prevalence,” and “incidence” in both Turkish and English.

**Figure 1:**
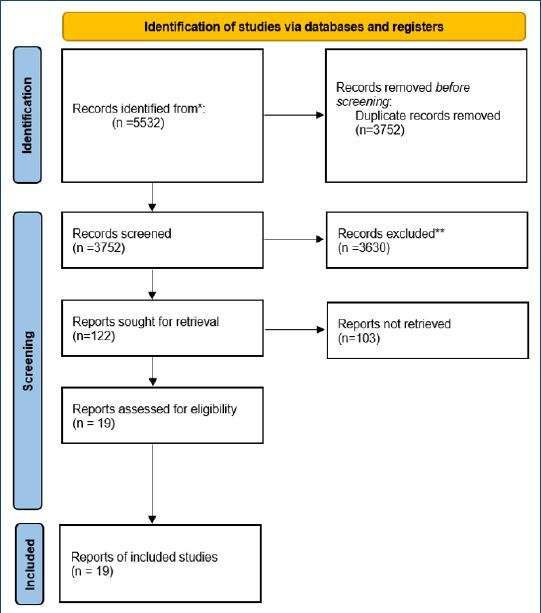
The Preferred Reporting Items for Systematic Reviews and Meta-Analyses flow chart.

Comprehensive prospective, retrospective, and cohort studies with full texts in English or Turkish were considered, excluding theses, conference papers, and reviews. The Preferred Reporting Items for Systematic Reviews and Meta-Analyses (PRISMA) flow chart (Figure 1) shows the screening process.

*All articles found in the literature search. **Articles that did not meet the inclusion criteria or whose full text was not available.

### Determining the eligibility criteria for articles

Meeting the criteria outlined above and selected after the exclusions indicated in Figure 1, the study was established based on the Patient, Intervention, Outcomes, Study Design (PICOS) model:

P-Patient: Women in the postpartum period
I-Intervention: UI
C-Comparison: Factors contributing to PUI and associated conditions
O-Outcomes: Incidence of PUI and contributing factors
S-Study design: Comprehensive prospective, retrospective, and cohort research articles reporting on the incidence, duration, and associated factors of PUI were included in the systematic review.

### Evaluation of the quality of studies

The quality of the 19 eligible studies was assessed using Polit and Beck’s criteria, which evaluate the study’s objectives, methodology, sample characteristics, findings, and discussion^16^. Each study was independently evaluated by both researchers to minimize errors. A score of “1 point” was assigned for each criterion met and “0 points” for unmet criteria. The average scores of the studies were then calculated.

### Data synthesis

After independent evaluations by both researchers, the study quality scores ranged from 9 to 12 (Table 1). A coding guideline was developed to input study results addressing the research questions. Data were analyzed in IBM SPSS V26, extracting descriptive characteristics, ratios, and median values of the factors investigated.

**Table 1:** Characteristics of studies.

Author/year	Type of stud	Sample size	UI evaluation time	Incidence of UI	The most common UI and rate	The average quality score of the studies
Lin et al., 2023^21^	Cohort	303	PP’s first 12 months	16.5%	SUI 16.5%	11
Elbiss and Abu-Zidan, 2023^22^	Prospective	101	PP’s first 3 months	13.8%	SUI 93.0%	11
Xu et al., 2023^23^	Retrospective	610	PP’s first 6–8 weeks	20.5%	SUI 20.5%	9
Urer et al., 2023^24^	Retrospective	142	PP’s first 3 months	39.4%	SUI 39.4%	11
Yang et al., 2022^25^	Cross-sectional	780	PP’s first 6–8 weeks	16.4%	SUI 11.1%	11
Ferrari et al., 2022^26^	Prospective	6,023	PP’s first 6 months	12.6%	SUI 58.2%	11
Löjdahl et al., 2022^27^	Prospective	13,670	PP’s first 3 months	52.0%	SUI 49.7%	12
Rajavuori et al., 2022^28^	Prospective	547	PP’s first 3 months	16.1%	Not specified	11
Diez-Itza et al., 2020^29^	Prospective	315	PP’s first 12 months	39.7%	SUI 39.7%	9
Åhlund et al., 2020^7^	Prospective	410	PP’s first 12 months	56.9%	SUI 45.4%	12
Johannessen et al., 2018^30^	Prospective	976	PP’s first 12 months	30.7%	Not specified	11
Chang et al., 2018^10^	Cohort	866	PP’s first 12 months	51.5%	SUI 51.5%	10
Fritel et al., 2016^31^	Cohort	2,002	PP’s first 6 months	14.0%	SUI 37.0%	10
Pizzoferrato et al., 2016^32^	Prospective	186	PP’s first 12 months	34.4%	SUI 24.8%	10
Brown et al., 2015^33^	Cohort	1,507	PP’s first 12 months	47.0%	Not specified	10
Mannion et al., 2015^34^	Cohort	1,574	PP’s first 12 months	49.0%	Not specified	11
Obioha et al., 2015^35^	Prospective	250	PP’s first 3 months	7.4%	SUI 7.4%	11
Ruiz Vinaspre Hernández et al., 2013^36^	Cohort	402	PP’s first 6 months	12.2%	Not specified	12
Yoshida et al., 2013^37^	Prospective	17	PP’s first 6 months	29.4%	SUI 29.4%	11

Note: P: postpartum; UI: urinary incontinence; SUI: stress urinary incontinence.

## RESULTS

### Characteristics of studies

The systematic review included studies from 2013 to 2023. Of these, 52.6% were prospective, 31.5% cohort, 10.5% retrospective, and 5.2% cross-sectional. The participants comprised women within the first year postpartum, irrespective of whether they experienced incontinence during or before pregnancy or after childbirth. The sample sizes ranged from 17 to 13,670, with a total of 30,681 participants across all studies (Table 1).

### Postpartum urinary incontinence results

Most studies were prospective, with a higher proportion evaluating UI within the first 12 months postpartum (42.1%; 8 studies). The reported incidence of PUI varied, ranging from 7.4 to 56.9%, with a median of 29.4%. PSUI was identified as the most common occurrence in 73.7% of the studies (14 studies), with reported rates ranging from 7.4 to 93.0%, and a median of 38.2%. Ten studies (52.6%) reported higher rates of PUI among those who gave birth vaginally, with PSUI rates ranging from 10.1 to 80.7%, and a median of 50.9%. Other reported risk factors included low maternal education level (median rate: 64.3%), multiparity (median rate: 39.0%), primiparity (median rate: 37.3%), and perineal laceration (median rate: 36.5%) (Table 2).

**Table 2:** Results of studies on postpartum urinary incontinence.

Results	Number of studies reviewed (n=19)	
	n	%
PUI evaluation time		
Studies evaluating the first 6–8 weeks	2	10.5%
Studies evaluating the first 3 months	5	26.3%
Studies evaluating the first 6 months	4	21.1%
Studies evaluating the first 12 months	8	42.1%
Types of PUI detected in studies		
Stress urinary incontinence (SUI)	14	73.7%
Study that detected incontinence but did not specify the type	5	26.3%
Studies reporting results in terms of factors that may cause PUI		
Vaginal delivery	10	52.6%
Cesarean section	9	47.3%
Age of mother <35	7	36.8%
Interventive delivery	6	31.5%
Obesity	6	31.5%
Primiparous	5	26.3%
Multiparous	4	21.1%
Episiotomy	4	21.1%
Prolonged second stage of labor	4	21.1%
Perineal laceration	4	21.1%
High head circumference of the newborn	3	15.7%
Low level of education	3	15.7%
According to the results of the study, the rates of the factors stated to cause PUI (in %)		
Factors	Min-max	Median
Vaginal delivery	10.1–80.7%	50.9%
Cesarean section	7.2-33.4%	13.9%
Age of mother <35	13.6-48.0%	15.8%
Interventive delivery	10.5-32.6%	18.8%
Obesity	10.7-88.0%	16.3%
Primiparous	13.0-100.0%	37.3%
Multiparous	14.5-70.4%	39.0%
Episiotomy	11.4-25.7%	20.6%
Prolonged second stage of labor	10.8-35.9%	17.1%
Perineal laceration	18.0-100.0%	36.5%
High head circumference of the newborn	8.3-67.8%	20.0%
Low level of education	11.1-77.7%	64.3%
PUI incidence rates stated in the study results (in %)		
PUI rate	7.4–56.9%	29.4%
The most common rates of PSUI in the study results (in %)		
PSUI rate	7.4–93.0%	38.2%

Note: P: postpartum; UI: urinary incontinence; SUI: stress urinary incontinence; PUI: postpartum urinary incontinence; PSUI: postpartum stress urinary ıncontinence.

## DISCUSSION

This systematic review aimed to determine the incidence and prevalence of UI in the first year postpartum, the predominant type of UI, and its associated factors by analyzing 19 studies. ICS recently reported a 21% prevalence of SUI in the first year after childbirth^17^. However, in Turkey, studies on PUI are limited. Our review found that the incidence of PUI ranged from 7.4 to 56.9%, with a median of 29.4%. SUI was identified as the most common type, with rates ranging from 7.4 to 93.0% and a median of 38.2%. These findings align with existing literature on PUI^3^ prevalence and the predominance of SUI^2^.

During childbirth, pelvic floor support can be compromised due to mechanical stress, injuries, and nerve damage, with a cesarean section before labor onset believed to reduce the risk of SUI^18^. Ten of the 19 studies in this review identified vaginal delivery as a significant risk factor for PUI, with rates from 10.1 to 80.7% and a median of 50.9%. Perineal laceration and episiotomy were also significant, with median rates of 36.5 and 20.6%, respectively. Vaginal delivery, episiotomy, perineal trauma, operative delivery, prolonged labor, and epidural analgesia are documented contributors to PUI^19^.

Multiparity and primiparity emerged as significant risk factors for PUI, with median rates of 39 and 37.3%, respectively, highlighting that all pregnant women are at risk. Some studies report primiparous women as being at higher risk than multiparous women^20^. Additionally, low education levels were identified as a risk factor in three studies, with rates from 11.1 to 77.7%. These findings emphasize the need for targeted interventions for women with lower education levels in prenatal and postnatal care to prevent and manage UI.

## CONCLUSION

The studies reviewed in this review reveal that UI affects approximately one in four women in the first year after birth, with SUI being the most common type. Vaginal delivery and related practices significantly increase the risk. These findings highlight the importance of perinatal, obstetric, and gynecological nurses in evaluating, diagnosing, preventing, and managing UI, often overlooked in postpartum care.

Implementing evidence-based practices in prenatal, intrapartum, and postnatal care is crucial. Holistic risk assessments for UI should be conducted for all pregnant and postpartum women. Nursing care plans should be tailored based on these assessments, and their effectiveness should be evaluated. By doing so, nurses can provide high-quality care and help develop care standards.

## References

[1] D’Ancona C, Haylen B, Oelke M, Abranches-Monteiro L, Arnold E, Goldman H (2019). The International Continence Society (ICS) report on the terminology for adult male lower urinary tract and pelvic floor symptoms and dysfunction. Neurourol Urodyn.

[2] Doumouchtsis SK, Tayrac R, Lee J, Daly O, Melendez-Munoz J, Lindo F (2022). An International Continence Society (ICS)/International Urogynecological Association (IUGA) joint report on the terminology for the assessment and management of obstetric pelvic floor disorders. Continence.

[3] Suar G, Cevik F, Simal Yavuz N, Ozerdogan N (2023). Urinary incontinence in the postpartum 1-year period: its prevalence and effect on psychosocial status of women. Low Urin Tract Symptoms.

[4] Fritel X, Fauconnier A, Levet C, Benifla JL (2004). Stress urinary incontinence 4 years after the first delivery: a retrospective cohort survey. Acta Obstetr Gynecol Scandinavica.

[5] Soave I, Scarani S, Mallozzi M, Nobili F, Marci R, Caserta D (2019). Pelvic floor muscle training for prevention and treatment of urinary incontinence during pregnancy and after childbirth and its effect on urinary system and supportive structures assessed by objective measurement techniques. Arch Gynecol Obstet.

[6] Xu C, Chi X, Guo Y, Chen Y, Chen X (2023). The association between the interdelivery interval and early postpartum urinary incontinence in women who had consecutive vaginal deliveries: a retrospective cohort study. Ann Transl Med.

[7] Åhlund S, Rothstein E, Rådestad I, Zwedberg S, Lindgren H (2020). Urinary incontinence after uncomplicated spontaneous vaginal birth in primiparous women during the first year after birth. Int Urogynecol J.

[8] Andrews V, Shelmeridine S, Sultan AH, Thakar R (2013). Anal and urinary incontinence 4 years after a vaginal delivery. Int Urogynecol J.

[9] Babini D, Lemos A (2020). Risk factors for urinary incontinence in primiparous adolescents after vaginal delivery: a cohort study. J Pediatr Adolesc Gynecol.

[10] Chang SR, Lin WA, Chang TC, Lin HH, Lee CN, Lin MI (2018). Risk factors for stress and urge urinary incontinence during pregnancy and the first year postpartum: a prospective longitudinal study. Int Urogynecol J.

[11] Gartland D, MacArthur C, Woolhouse H, McDonald E, Brown SJ (2016). Frequency, severity and risk factors for urinary and faecal incontinence at 4 years postpartum: a prospective cohort. BJOG Int J Obstetr Gynaecol.

[12] Kristiansson P, Samuelsson E, Schoultz B, Svärdsudd K (2001). Reproductive hormones and stress urinary incontinence in pregnancy. Acta Obstet Gynecol Scand.

[13] Wang X, Xu X, Luo J, Chen Z, Feng S (2020). Effect of app-based audio guidance pelvic floor muscle training on treatment of stress urinary incontinence in primiparas: a randomized controlled trial. Int J Nurs Stud.

[14] Liang Y, Chen Y, Yu X, Li X (2021). Quality of life among women with postpartum urinary incontinence: a cross-sectional study. Gynecol Obstetri Clin Med.

[15] Kamioka H (2019). Preferred reporting items for systematic review and meta-analysis protocols (prisma-p) 2015 statement. Japanese Pharmacol Therapeutics.

[16] Polit DF, Beck CT (2017). Nursing research: generating and assessing evidence for nursing practice.

[17] Jansson M, Franzen K, Tegerstedt G, Hiyoshi A, Nilsson K (2021). Postpartum urinary incontinence types, prevalence, and risk factors before, during, and after pregnancy and childbirth: a prospective cohort study. ICS Online.

[18] Kocaöz S, Kafiye E (2009). Gebelik ve vajinal doğum sonrasi dönemde stres üriner inkontinansin önlenmesinde konservatif tedavi yöntemleri ve hemşirenin rolleri. Turkiye Klinikleri J Nurs Sci.

[19] Tähtinen RM, Cartwright R, Vernooij RWM, Rortveit G, Hunskaar S, Guyatt G H (2019). Long-term risks of stress and urgency urinary incontinence after different vaginal delivery modes. Am J Obstetr Gynecol.

[20] Gupta A, Pampapati V, Khare C, Murugesan R, Nayak D, Keepanasseril A (2021). Postpartum urinary retention in women undergoing instrumental delivery: a cross-sectional analytical study. Acta Obstet Gynecol Scand.

[21] Lin YH, Chang SD, Hsieh WC, Chang YL, Chueh HY, Chao AS (2018). Persistent stress urinary incontinence during pregnancy and one year after delivery; its prevalence, risk factors and impact on quality of life in Taiwanese women: an observational cohort study. Taiwan J Obstet Gynecol.

[22] Elbiss HM, Abu-Zidan FM (2023). Postpartum urinary incontinence of nulliparous women: a prospective cohort study. Medicine.

[23] Xu C, Guo Y, Chi X, Chen Y, Chu L, Chen X (2023). Establishment and validation of a simple nomogram for predicting early postpartum stress urinary incontinence among women with vaginal delivery: a retrospective study. BMC Women’s Health.

